# An Analysis of Adult Obesity and Hypertension in Appalachia

**DOI:** 10.5539/gjhs.v5n3p127

**Published:** 2013-02-22

**Authors:** Janaranjana Herath, Cheryl Brown

**Affiliations:** 1 Tillman School of Business, Mount Olive College, Mount Olive, USA; 2 Davis College of Agriculture, Natural Resources and Design, West Virginia University, Morgantown, USA

**Keywords:** Appalachia, hypertension, Logit analysis, obesity, simultaneous equations

## Abstract

Obesity is a major health problem in the United States, and the burden associated is high. Hypertension seems to be the most common obesity-related health problem. Studies show that hypertension is approximately twice as prevalent among the obese as in the non-obese population. This study has two main objectives. First, to examine the association between obesity and hypertension within the context of economic growth in Appalachia, and second to estimate the cost of hypertension linked to obesity in Appalachia. The study uses simultaneous equations and Logit analysis for estimations. Data are from Behavior Risk Factor Surveillance Systems (BRFSS) surveys of 2001 and 2009. Results for simultaneous analysis show that hypertension decreases with decreasing obesity, increasing employment growth, and increasing income. Logit analysis highlights the importance of controlling obesity, income growth, employment growth, education, and exercises in mitigating hypertension in Appalachia. Ageing indicates a high potential of getting hypertension. Total economic cost of hypertension linked to obesity is $9.35 billion, significantly a high cost to Appalachia. Overall, results reveal the impacts of obesity on hypertension and some possible ways of mitigation. Thus, results suggest a comprehensive set of policies to Appalachia which sufficiently improve employment opportunities, educational facilities, and healthcare facilities with adequately addressed to obesity and hypertension.

## 1. Introduction

In the United States, obesity is a major health problem, and approximately 34 percent of the U.S. adult population is obese (Sabate & Wien, 2010; Finkelstein et al., 2009). With the current trend of obesity, 50 percent of the U.S. population will be obese in 2030 (Dor et al., 2010). Diseases, like heart disease, diabetes (type II), hypertension, cancer, arthritis, asthma, and some psychological disorders are linked with obesity (Sturm et al., 2004; Malnick & Knobler, 2006; Miljkovic & Nganje, 2008). Obesity increases the risk of premature mortality (WHO, 2005) and nearly 300,000 annual deaths are associated with obesity in the United States (Miljkovic & Nganje, 2008). The magnitude of health impacts depends on the levels of obesity-related diseases, socioeconomic and behavioral characteristics of individuals (Cawley et al., 2005; Sacerdote, 2007), and environmental and geographical characteristics (Inagmi et al., 2006; Wang et al., 2007) of a particular region.

Obesity is an established risk factor for hypertension. Hypertension seems to be the most common obesity-related health problem (Kurukulasuriya et al., 2011). Studies show that hypertension is approximately twice as prevalent among the obese as in the non-obese population (Flegal et al., 2002; Ogden et al., 2002). The prevalence of obesity-related hypertension varies with age, race, and gender of the population studied (Sung et al., 2012). Minority groups show a high prevalence of undiagnosed and uncontrolled hypertension (Gillespie, 2011).

The economic burden associated with obesity is high. Hammond and Levine (2010) identified four major categories of economic impacts associated with obesity: direct medical costs, productivity costs, transportation costs, and human capital costs. According to the U.S. Department of Health and Human Services (2010), the annual cost of obesity was $147 billion in 2008 and people who were obese had annual medical costs that were $1,429 higher than the cost for people of normal body weight. The overall annual cost of being obese is $2,646 for an obese man and $4,879 for an obese woman (Dor et al., 2010).

The study has two main objectives. First, to examine the association between obesity and hypertension within the context of economic growth in Appalachia for the decade from 2000 to 2010, and second to estimate the cost of hypertension linked to obesity in Appalachia.

The paper is organized into six sections. Section 2 provides background information of Appalachia. Section 3 explains methodology and data sources. Section 4 discusses empirical results and analysis. Section 5 presents conclusions and policy implications.

## 2. Background Information of the Appalachian Region

The Appalachian Region consists of 420 counties in 13 states: New York, Pennsylvania, Ohio, Maryland, West Virginia, Kentucky, Virginia, North Carolina, Tennessee, South Carolina, Georgia, Alabama, and Mississippi. In 2009, Appalachian Regional Commission (ARC) classified the region into five sub-regions: Northern, North Central, Central, South Central, and Southern. The region’s economy is highly dependent on mining, forestry, agriculture, chemical industries, professional service, and manufacturing (ARC, 2011). Ninety-six Appalachian counties were considered economically distressed in 2011. Ninety counties are at risk and 219 have transitional economies. Central Appalachia exhibits high economic distress with high poverty, poor healthcare services, and high educational disparities (ARC, 2011). The region is facing a lack of human, financial, and technical resources due to its geographic isolation, disproportionate social and economic distress, low household incomes, and a declining tax base.

The Appalachian region is home for nearly 24.8 million people, and its population growth from 2000 to 2008 was slower than the national rate (ARC, 2010). The highest population is reported in Northern Appalachia while the lowest is in Central Appalachia. In many parts of Appalachia young people are moving out and retirees are moving in (ARC, 2010). According to the economic overview of Appalachia (ARC, 2011), unemployment rates in two thirds of Appalachian counties are higher than the national rate. The average unemployment rate is 9.7 percent, which is 0.4 percent higher than the 2009 national rate. Central, Southern and South Central Appalachia have an unemployment rate greater than 11 percent. Per capita personal income, average earnings, and per capita investment income are lower than national averages. According to the economic assessment of Appalachia (2010), educational levels in the region are low, and all parts of the Appalachian region lag behind the nation in college attendance and completion. Among sub-regions Central Appalachia reports the lowest educational attainment.

As a whole, Appalachia reports higher rates of serious disease and mortality rates than national levels (ARC, 2010). Nearly 44 percent of the Appalachian population is obese with the highest rate reported in southeast Appalachia (Wewers et al., 2006). Counties on the “high end” of obesity prevalence are Dallas County (41.6 percent) and Greene County (43.7 percent) in Alabama; and Holmes County (42.6 percent), Humphreys County (41.9 percent), and Jefferson County (41.3 percent) in Mississippi.

## 3. Estimation Methods

Within the consumer’s utility maximization context, the study uses simultaneous equations and Logit analysis, to examine the association between obesity and hypertension within the context of economic growth, and to calculate the cost of obesity-related hypertension.

### 3.1 Examine the Association between Obesity and Hypertension

A system of simultaneous equations was used with county level data to examine the relationship between obesity, hypertension, income growth, and employment growth in Appalachian region for the period of 2000 to 2010, the last decade. The model is derived based on the simultaneous approach studies of Carlino and Mills (1987), Deller et al. (2001) and Rosenberger et al. (2005). A system of equations estimates all the identified structural equations together as a set. A Simultaneous approach accounts for interactions among the interdependent variables which gives comprehensive estimations. Simultaneity helps in overcoming inconsistency and bias, and leads to asymptotically efficient estimations with three-stage least squares (3SLS) analysis. 3SLS takes into account the contemporaneous correlation among error terms. However, 3SLS is more sensitive to the model specification error, which can be overcome by checking results with 2SLS estimations.

Income, employment, obesity, and hypertension are interdependent. Thus, a model with a system of simultaneous equations is used to give better results than a single equation approach. As the intention of this objective is to examine the impacts at county level, county average values of income, employment, obesity, and hypertension are used in the model. Also, all other variables of social factors (SF), behavioral factors (BF) and environmental factors (EF) are applied at county level.

The variables Income*, Employment*, Obesity*, and Hypertension* represent the equilibrium levels of income, employment, obesity, and hypertension. Ω^I^, Ω^E^, Ω^O^, and Ω^H^ are a set of variables describing initial conditions that measure social factors (SF), environmental factors (EF), and behavioral factors (BF) that are linked to obesity-related health implications. Thus, the general form of the four equations model is:


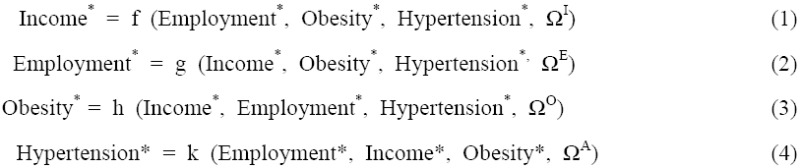


From the equilibrium framework of the model, a simple liner relationship among the variables can be presented as (where I is income, E is employment, O is obesity, and H is hypertension):


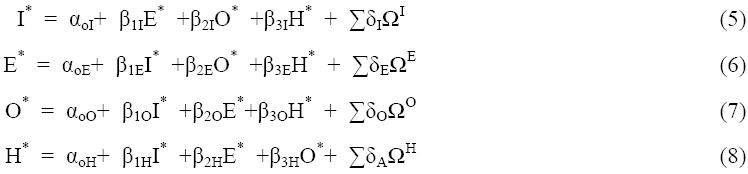


where α values indicate the intercepts of each equation, β indicates coefficient estimations of each interdependent variable, and δ indicates the coefficients of the set of variables that describe initial conditions.

Moreover, income, employment, obesity, and hypertension likely adjust to their equilibrium levels with substantial lags (i.e., initial conditions). Thus, partial adjustment equations to the equilibrium levels are as:


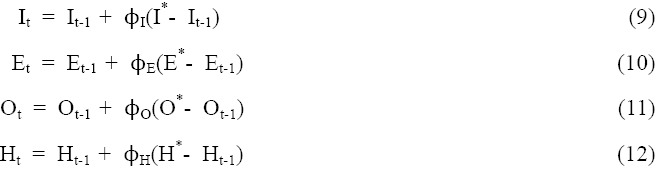


The current, income, employment, obesity, and hypertension level at time t are functions of their initial conditions, change between the equilibrium values and initial conditions, and their respective speed of adjustment values. I_t-1_, E_t-1_, O_t-1_ and H_t-1_ are initial conditions of income, employment, obesity, and arthritis. ϕ_I_, ϕ_E,_ ϕ_O,_ and ϕ_H_ are the speed of adjustment coefficients related to the desired utility maximization level of income, employment, obesity, and hypertension. Substituting Equations 13 through 16 into Equations 9 through 12, and rearranging the model can be expressed as:


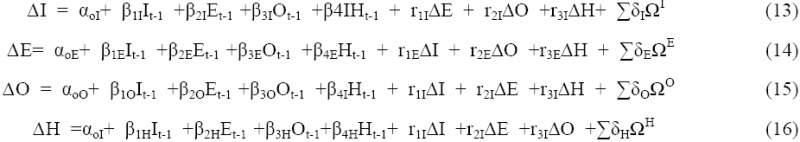


where ∆I, ∆E, *∆*O, and ∆H are the changes in income, employment, obesity, and hypertension, respectively. The speed of adjustment coefficients become embedded in the linear estimated parameters α, β, r and δ. The model captures structural relationships while simultaneously isolating the influence of obesity on public health. Equations 13-16 estimate short-term adjustments of income, employment, obesity and hypertension (∆I, ∆E, *∆*O, and ∆H) to their long-term equilibriums (I*, E*, O*, and H*).

### 3.2 Estimate the Cost of Hypertension Linked to Obesity in Appalachia

To accomplish this objective Logit analysis of a response function (Equation 17) is used with the total expenditures for hypertension. Individual level data for 2009 related to hypertension are used for the analysis.

A Logit analysis of a response function for hypertension with obesity as a qualitative exogenous variable would give the coefficient for the marginal impact of obesity for hypertension. If the Logit equation is perfectly defined, this coefficient for obesity indicates the contribution of obesity to hypertension. Thus, multiplying this value by the known healthcare expenditures of hypertension would give the cost of hypertension linked to obesity. These types of dose-response functions are common in cost calculations (Quah & Boon, 2002; Srivastava & Kumar, 2001; Lvovsky, 1998; Zuidema & Nentjes, 1997; Ostro, 1995).

Suppose, H_i_ represents the hypertension of the i^th^ individual, which appears as a qualitative dependent variable equal to one if the individual has the disease and zero otherwise. E_i_ is equal to one if the i^th^ individual is employed, and O_i_ is equal to one if the i^th^ individual is obese. I_i_ is household income for the i^th^ individual. The variables that represent socioeconomic factors (SF), behavioral factors (BF), and environmental factors (EF) are specified for each individual.


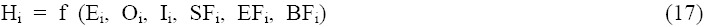


The marginal effect of the estimated equation can be expressed as:


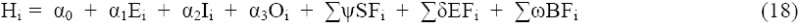


where α_0_ indicates the intercept of the equation, α_1,_ α_2,_ and α_3_ are coefficient estimations of E_i_, O_i_, and I_i_. The summations of the coefficients of SF, EF and BF are indicated by ψ, δ, and ω.

To obtain the total economic cost (TEC_H_) of obesity related to hypertension, the total expenditures on healthcare for hypertension (THE_H_) in Appalachia is multiplied by the coefficient of O_i_ which is α_3_ from the marginal effects of the Logit equation for hypertension.


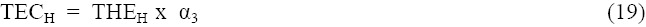


However, the resulting total economic cost would not account for any loss in productivity due to absenteeism or the loss to an individual over his/her lifetime of lost income.

### 3.3 Types and Sources of Data

Behavior Risk Factor Surveillance Systems (BRFSS) survey data for years 2001 and 2009 are the main source of data. In examining impacts of obesity on hypertension, county level data of both 2001 and 2009 are used. Individual level data of 2009 are used in estimating the cost of hypertension linked to obesity. BRFSS is a survey of health risk behaviors in non-institutionalized civilian adults, age 18 years and over. These data were collected from a stratified random sample through computer-assisted telephone interviewing by state health departments with the collaboration of the Center for Disease Control and Prevention (CDC). Data for the county level employment, income, number of adults, and population, are collected from Bureau of Economic Analysis (BEA, 2009). The U.S. Census Reports (2000 and 2010) and National Health and Nutritional Examination Survey [NHANES] (2009) also are referred as necessary.

## 4. Empirical Results and Discussion

Descriptions of variables used for estimation are presented in Tables 1 and 2. Table 1 shows county-level descriptive statistics of variables for all Appalachian counties for 2001 and 2009. Table 2 indicates county-level descriptive statistics for behavioral and environmental variables for all Appalachian counties for 2009.

**Table 1:** County-level descriptive statistics of variables for all Appalachian counties, 2001, 2009

Variable	Description	Mean	Std. Dev.
INC01	Average annual household income	$26,616	$5,617
EMP01	Number of adults 18 and older who were employed	26,481	45,891
OBE01	Percentage of obese adults 18 and older	23.7	3.5
HYP01	Percentage of adults 18 and older with hypertension	29.7	4.6
INC09	Average annual household income	$37,460	$7,849
EMP09	Number of adults 18 and older who were employed	26,091	45,176
OBE09	Percentage of obese adults 18 and older	30.8	5.4
HYP09	Percentage of adults 18 and older with hypertension	42.1	9.5
POP	County population in 2009	59,399	96,730
AGE	Average age of adults 18 and older in 2009	54.9	4.5
MARED	Percentage of population that was married in 2009	56.0	10.0
EDU	Percentage of population that has had some college, completed a college degree or has a professional or graduate degree in 2009	44.2	12.3
MALE	Percentage of males in 2009	36.8	8.7

**Table 2:** County-level descriptive statistics for behavioral and environmental variables for all Appalachian counties, 2009

Variable	Description	Mean	Std. Dev.
SMOKE	Percentage of county's population 18 and older who smoke	24.0	9.2
DRINK	Percentage of county's population 18 and older who consume alcohol	30.7	16.6
SLEEP	Average number of sleepless days of an adult 18 and older	8.5	2.4
GDHLTH	Percentage of county's population 18 and older that reported having good health	71.6	12.6
HCRPLN	Percentage of county's population 18 and older that reported having a healthcare plan	85.6	7.7
EXECISE	Average total minutes of exercise per week by an adult 18 and older	368.8	157.3
NORTH	1 if county is in northern regions of Appalachia; 0 otherwise	35.5	47.9
HCRFAC	Access to healthcare facilities (per 100,000 population)	52.0	24.1
RECRE	Access to recreation facilities (per 100,000 population)	6.5	4.9

### 4.1 Determining the Impacts of Obesity on Hypertension in the Appalachian Region

A system of simultaneous equations was used to determine the impacts of obesity on hypertension in Appalachia. Results of the three stage least squares analyses are presented in Table 4.1.6. The first column shows the variables used for analysis. Columns 2 and 3 indicate results for the income change (INCC) equation while columns 4 and 5 show results for the employment change (EMPC) equation. The results for the obesity change (OBEC) equation are shown in columns 6 and 7. The last two columns in the table show results for the hypertension change (HYPC) equation.

The empirical results for the income change equation indicate that income change (INCC) is significantly and positively related to employment change (EMPC), that a one percent increase in employment growth increases income growth by 0.8 percent. Hypertension change and income change are significantly and positively related, when hypertension change increases by one percent income change increases by 0.33 percent. The coefficient of the initial value of hypertension (HYP01) indicates that counties that reported higher hypertension rates in 2001 had higher rates of income growth compared to other counties. This result is supported by Lee et al. (2009) who found that hypertension has increased in all income groups: by 85 percent in the lowest income group, by 80 percent in the lower middle income group, by 91 percent in the upper middle income group and by 117 percent in the highest income group. Initial values of income (INC01) and employment (EMP01) are significant but the impacts are minimal. A more educated county population (EDU) means income growth will be higher. A significant and positive value for NORTH means that northern Appalachia has had higher income growth compared to southern Appalachia, as reported by the ARC (2010).

The empirical results for employment change (EMPC) indicate that the initial value of income (INC01) increases employment growth at the county level. The significant and positive results for education show that a one percent increase in education rates increases employment growth by 0.2 percent. The significant and negative relationship between MALE and employment growth indicates that females contributed more to employment growth from 2001 to 2009. This could be associated with creation of more jobs for females in the healthcare sector.

The empirical results for obesity change (OBEC) indicate that income change and obesity change are significantly and positively related. Generally, higher income at the individual level is associated with lower obesity (Chou et al., 2004). The counterintuitive result found here is supported by Ewing et al. (2003), Loureiro and Nayga (2004), and Rosin (2008) who found that weight gain increased with urbanization and development and related increases in income. The significant and positive relationship of hypertension change and obesity change implies that a one percent increase in hypertension change increases obesity change by 0.9 percent. This result is also supported by the findings of Lee et al. (2009). A significant result for the initial hypertension rate indicates that counties that reported a high percentage of its population with high blood pressure in 2001 had higher rates of obesity growth compared to others. The initial income level has a significant and negative relationship with obesity change but the impact is minimal. While smoking has positive impacts on obesity growth rates, drinking was found to have negative impacts; both impacts are minimal. Smoking was expected to have a negative relationship with obesity; however, Peters et al. (2003) found that the relationship between smoking and overweight is not widely understood.

Results for hypertension change (HYPC) show that employment change and hypertension change are significantly and negatively related; when employment change increases by one percent, hypertension change decreases by 1.1 percent. This implies that the greater the employment opportunities, the lower the growth of high blood pressure. This result is supported by Brackbill et al. (1995), who reveal lower rates of hypertension with more employment opportunities. Obesity change and hypertension change have a positive and significant relationship; when obesity change increases by one percent hypertension change increases by 0.7 percent. This outcome is supported by the findings of Kotchen (2008), which shows obesity leads to high rates of hypertension. The initial obesity rate (2001) also has a significant and positive relationship with hypertension change. Results show that age increases hypertension growth in Appalachian counties. It is common that high blood pressure increases in men after the age of 35 and women after the age of 50 (Stibich, 2007). Results show that smoking reduces hypertension growth, and drinking increases hypertension growth at the county level. According to medical research, smoking can increase hypertension of an individual (Rosen et al., 2006) and too much alcohol consumption can affect hypertension negatively. Populations living in northern Appalachia have less growth in hypertension compared to those in southern Appalachia. This may be due to better socioeconomic conditions in the north compared to southern parts of Appalachia.

**Table 3:** Results for system of equations including change in hypertension (HYPC)

Variable	Income Change		Employment Change		Obesity Change		Hypertension Change	

	Coefficient	P>|Z|	Coefficient	P>|Z|	Coefficient	P>|Z|	Coefficient	P>|Z|
INCC			0.1883	0.13	0.9314***	0.01	0.1625	0.58
EMPC	0.3379**	0.08			0.5318	0.20	-1.1478***	0.00
OBEC	0.0813	0.29	-0.0905	0.27			0.7067***	0.00
HYPC	0.3390 *	0.01	-0.0797	0.30	0.9091***	0.00		
INC01	-0.0001***	0.00	0.0001***	0.01	0.0001***	0.00	-0.0001	0.31
EMP01	0.0001**	0.06	-0.0001	0.29	-0.0001	0.56	-0.0001	0.79
OBE01	0.1743	0.62	-0.4679	0.19	-3.9204***	0.00	2.8437***	0.00
HYP01	1.1125***	0.00	-0.0583	0.83	2.5085***	0.00	-2.8371***	0.00
AGE	-0.0016	0.53			-0.0086	0.17	0.0062***	0.02
EDU	0.0012**	0.07	0.0021***	0.00				
MALE			-0.0022***	0.00				
MARED	0.0006	0.35						
EXECISE					0.0001	0.85		
HCRPLN			-0.0005	0.55				
SLEEP							-0.0031	0.44
SMOKE					0.0029**	0.07	-0.0021**	0.03
DRINK					-0.0051***	0.00	0.0042***	0.00
NORTH	0.0731***	0.00					-0.0659 ***	0.00

Number of observations = 420. R^2^ values: INCC = 0.86; EMPC = 0.10; OBEC = 0.04; HYPC = 0.39. Chi^2^ values: INCC = 2093.54; EMPC = 77.96; OBEC = 170.75; HYPC = 883.81.

***, **, * *are significant at* 1%, 5%* and* 10%* respectively*.

### 4.2 Estimate the Cost of Hypertension Linked to Obesity in Appalachia

To estimate costs of hypertension linked to obesity, the result of Logit analysis was applied to total expenditure on hypertension in the Appalachian region. The result from the Logit analysis supplies the probability of an individual having hypertension, which is used to estimate the cost of hypertension.

### 4.3 Logit Analysis

For Logit analysis, individual data were used for hypertension, after removing data for individuals who were pregnant or who had any kind of missing data of exogenous variables, like income, BMI, or education. Thus, the sample size was around 22,000 individuals for all of Appalachia. The presence of hypertension was the dependent variable. For each individual the presence of obesity, age, marital status, education level, employment status, annual household income, gender, and race were the socioeconomic variables used. Among behavioral factors, sleepless days in the last month (Sleep), if the individual consumes alcohol (Drinks), if the individual smokes (Smokes), and total minutes an individual engaged in exercise in the previous week (Exercise) were used. Descriptive statistics of the variables used in the Logit analysis are presented in Table 4.

**Table 4:** Descriptive statistics of the variables for 2009, used for Logit analysis

Variable	Description and unit	Mean	Std. Dev.
Hypertension	1if has hypertension; 0 otherwise	0.43	0.49
Obesity	1 if obese; 0 otherwise	0.31	0.46
Age	In years	55.46	16.06
Marital status	1 if married; 0 otherwise	0.56	0.49
Education	1 if some college or higher; 0 otherwise	0.51	0.49
Employment	1 if employed; 0 otherwise	0.40	0. 49
Income	Annual income in dollars	$40,774	$24,815
Gender	1 if male; 0 if female	0.38	0.48
Race	1 if white; 0 if race other than white	0.90	0.29
Sleep	Number of sleepless days in previous month	8.50	10.56
Drinks	1 if drinks alcohol; 0 otherwise	0.34	0.47
Smokes	1 if smokes; 0 otherwise	0.21	0.41
Exercise	Number of minutes engaged in exercise for the previous week	403.57	674.95

Logit analysis for hypertension in Table 5 shows that an obese person is 23 percent more likely to get hypertension than a non-obese person. Some studies show that hypertension is approximately twice as prevalent among the obese as in non-obese people (Flegal et al., 2002; Ogden et al., 2002). Ageing of an individual increases the hypertension. If age increases by one year, the probability of getting hypertension increases by 1.1 percent. Education decreases the potential for getting hypertension. This may be due to better attention to healthcare with better education. If an individual is employed, s/he is 8 percent less likely to get high blood pressure. Even though the impact is low, an increase in income decreases the potential of getting hypertension, as does an individual’s positive employment status. A significant result for gender indicates that adult men have a higher probability (5 percent) of getting hypertension compared to adult women. This result is supported by McMahon et al. (1984) which showed that about 30 percent of hypertension cases are attributable to obese men. White individuals are less likely to get hypertension compared to individuals of other races; studies of African-Americans found the same results (Martins & Norris, 2004).

**Table 5:** Logit regression results: marginal effects for hypertension

Variable	Marginal Effect	Std. Err	P>|z|
Obesity	0.23481***	0.0081	0.00
Age	0.01121***	0.0003	0.00
Marital status	-0.01450*	0.0086	0.09
Education	-0.04972***	0.0083	0.00
Employment	-0.07721***	0.0088	0.00
Income	-0.00002***	0.0000	0.00
Gender	0.05112***	0.0079	0.00
Race	-0.12674***	0.0132	0.00
Exercise	-0.00004***	0.0001	0.00
Drinks	-0.00474	0.0084	0.57
Smokes	0.00471	0.0098	0.62

Number of observations = 20,706. LR chi^2^(12) = 4015.07; Prob > chi^2^ = 0.0000. Log likelihood = -2122.86; Pseudo R^2^ = 0.1421.

***, **, * *are significant at* 1%*,* 5%* and* 10%* respectively*.

### 4.3 Calculating Healthcare Expenditures for Hypertension (THE_A_)

Data for total healthcare expenditures for hypertension is limited. Thus, the calculation healthcare expenditures of hypertension for Appalachia is based on estimations of the Milken Institute (2007), who calculated treatment costs as well as productivity lost due to chronic diseases. Based on the Medical Expenditure Panel Survey (MEPS), National Household Education Survey (NHES) and other data from 2003 the Milken Institute projects costs for hypertension up to 2023 for each state in the United States. The estimations of the Milken Institute for 2009 were used to calculate the cost of hypertension for the Appalachian region.

The calculations are shown in Table 6. The first column presents the Appalachian states, the second column shows the total population in those states, the third column gives the total population in only the Appalachian counties of each state, and the fourth column shows the cost of hypertension for each state according to the estimations of the Milken Institute (2007). The fifth column shows the costs of hypertension for Appalachia, which were calculated by multiplying the costs of hypertension from column 3, by the percentage of the population in Appalachian counties of each state. Thus, the total cost of hypertension for the Appalachian region is $39.85 billion. As hypertension is not prevalent among children, the total calculated cost is assumed to be the total cost of hypertension for adults in Appalachia.

**Table 6:** Calculation of cost of hypertension for the Appalachian region ($billion), 2009

Appalachian States	Total Population in State	Total Population in Appalachian Counties	Cost of Hypertension ($billion)*	Cost for Hypertension in Appalachia ($billion)*
Alabama	4,779,736	3,024,719	8.95	5.66
Georgia	9,687,653	2,924,921	15.15	4.57
Kentucky	4,339,367	1,194,500	7.38	2.03
Maryland	5,773,552	247,997	8.28	0.36
Mississippi	2,967,297	623,260	5.82	1.22
New York	19,378,102	1,049,686	29.65	1.61
North Carolina	9,535,483	1,662,282	15.15	2.64
Ohio	11,536,504	2,013,203	18.12	3.16
Pennsylvania	12,702,379	5,736,617	19.84	8.96
South Carolina	4,625,364	1,167,523	1.20	0.31
Tennessee	6,346,105	2,801,826	10.82	4.78
Virginia	8,001,024	681,686	11.05	0.94
West Virginia	1,819,777	1,819,777	3.62	3.62
Total cost of hypertension for Appalachia				39.86

* Calculated by author.

Sources: U.S. Census Bureau (2010) and Milken Institute (2007).

### 4.4 Total economic cost of obesity-related hypertension (TEC_H_)

To obtain the total economic cost (TEC_H_) of obesity-related hypertension, the total healthcare expenditures for hypertension (THE_H_) (i.e. $39.86 billion) is multiplied by the coefficient of obesity (O_i_) (i.e. 0234), which was estimated using the marginal effects from the Logit analysis for hypertension. Thus, the total economic cost of hypertension (TEC_H_) is $9.35 billion. This is the total economic cost of hypertension linked to obesity in Appalachia.

## 5. Conclusions and Policy Suggestions

The county level estimation for hypertension in Appalachia for the period of 2001-2009 shows an increasing trend of hypertension with increasing obesity or vise versa. Higher growth of income has increased obesity growth, which increases hypertension subsequently. Growth of employment has decreased the growth of hypertension. These results highlight the changes of obesity, hypertension, income and employment within the period of 2001-2009. Results emphasize the lack of healthcare development parallel to income and employment growth in Appalachia, which could capture health benefits associated with economic growth.

Logit analysis based on individual level data highlights the importance of controlling obesity, income growth, employment growth, education, and exercises in mitigating hypertension in Appalachia. Ageing increases the potential of getting hypertension. Total economic cost estimation for hypertension linked to obesity is $9.35 billion, indicates a significant impact of obesity on hypertension in Appalachia.

Overall, results reveal the impacts of obesity on hypertension and possible ways of mitigation. Thus, results suggest a comprehensive set of policies to Appalachia which sufficiently improve employment opportunities, educational facilities, and healthcare facilities with adequately addressed to obesity and hypertension. As the Appalachian region is lack of infrastructure to take full advantage of emerging economic gains (ARC, 2010) any policy intervention aimed at health improvement and/or obesity reduction without physical infrastructure development will be less effective, especially in economically distressed counties.
